# Urban Ecosystem Health Assessment: Perspectives and Chinese Practice

**DOI:** 10.3390/ijerph10115874

**Published:** 2013-11-06

**Authors:** Meirong Su, Yan Zhang, Gengyuan Liu, Linyu Xu, Lixiao Zhang, Zhifeng Yang

**Affiliations:** State Key Joint Laboratory of Environment Simulation and Pollution Control, School of Environment, Beijing Normal University, Beijing 100875, China; E-Mails: sumr@bnu.edu.cn (M.S.); yzhang@bnu.edu.cn (Y.Z.); liugengyuan@bnu.edu.cn (G.L.); xly@bnu.edu.cn (L.X.); zhanglixiao@bnu.edu.cn (L.Z.)

**Keywords:** urban ecosystem health, evaluation framework, assessment method, Chinese practice

## Abstract

The concept of ecosystem health is a way to assess the holistic operations and development potential of urban ecosystems. Accelerated by the practical need for integrated ecosystem management, assessment of urban ecosystem health has been greatly developed and extensively applied in urban planning and management. Development is aimed at comprehensively evaluating the performance of urban ecosystems, identifying the limiting factors, and providing suggestions for urban regulation. The time has come for reviewing and establishing an instructional framework for urban ecosystem health assessment to shed light on certain essential issues of urban ecosystem health. Based on literature reviews and series of practice, a holistic framework of urban ecosystem health assessment is proposed. The framework covers the essential elements of urban ecosystem health and integrates three dimensions: theoretical foundation, assessment method, and practical application. Concrete assessment methods are also established, focusing on both external performance and internal metabolic processes. The practice of urban ecosystem health assessment in China is illustrated to briefly demonstrate the application of the established framework and methods. Some prospects are discussed for urban ecosystem health assessment and its application in urban planning and management.

## 1. Introduction

As a socioeconomic-ecological complex system [1], an urban ecosystem consists of residents and their environment in certain time and space scales, in which, ecologically-speaking, consumers are the dominant component lacking producers and decomposers [2]. The incomplete structure makes the urban ecosystem dependent and fragile, which is further aggravated by huge demand of resources for industrial production and human consumption as well as pollutant emissions. When the ecosystem stress is within the ecosystem’s regenerative capacity, it can self-restore. However, as more intensive human activities result in adverse environmental changes that jeopardize sustainability and impair ecological functions and societal services [3,4], more and more attention has been paid to the holistic performance and development potential of urban ecosystems. Disturbed by various visible environmental problems such as water shortage, air pollution, and land degradation, there is concern about whether the urban ecosystem can operate sufficiently well to support dense population, provide sustainable services, and maintain good environmental quality. Therefore, urban ecosystem health-combining the urban ecosystem’s ability to maintain its own renewal and self-generative capacity and satisfy reasonable demand from human society became a scientific topic [2] and was greatly driven by the extensive public concerns and decades of progress in ecosystem health research [5,6,7,8,9,10,11]. Urban ecosystem health integrates ecological, economic, social and human health factors and including not only the health and integrity of the natural and built environment, but also health of urban resident and whole society [2,12,13,14,15].

Because of its holistic perspective and applicability for the public and managers, the concept of urban ecosystem health has been applied extensively in practical urban planning and management [16]. Particularly, the assessment of the state of urban ecosystem health has been conducted to comprehensively measure the operation of urban ecosystems, to identify the limiting factors, and to provide suggestions for urban regulation. Accelerated by practical demands, science has provided a foundation for the development of assessment methods for urban ecosystem health, which include assessment indicators and mathematical models.

Multiple indicators based on different conceptual frameworks have been proposed to abstract information from the complicated urban system and to connect the theoretical background with practical management requirements [1,17]. Harpham established urban health indicators from economics, environment, public health, health expenditure, and nutrition points of view [18]. Based on the Healthy Cities Project, a global movement that puts health high on the social, economic and political agenda of city governments and promotes systematic policy for health, the World Health Organization built healthy urban ecosystem indicators covering fields of urban infrastructure, environmental quality, living environment, health care, education, *etc.* [19,20]. Guo *et al.* established urban ecosystem health indicators [21] in the framework of vigor, structure, resilience, ecosystem services, and population health by referring to the classic framework from Mageau *et al.* [22] and Rapport *et al.* [23]. Others set up indicators to represent the health situation of multiple subsystems [24,25]. While these indicators are established from different focuses and foundations, a unified conceptual model is still necessary to organize multiple factors in a holistic way.

Additionally, many mathematical models are used to treat and process the indicator data to satisfy a health assessment. One approach to these models is based on urban ecosystem health characteristics like fuzziness, hierarchy, and multiple-attributes. Examples of the methods are the fuzzy synthetic assessment model [21,26], fuzzy optimal assessment model [25], attribute theory model [27], and relative vector comprehensive assessment model [28]. Another approach taken by a few models is to focus on emergent problems during the process of urban ecosystem health assessment, e.g., unascertained measure model [29], matter element model [30], and projection pursuit model [31]. Although these methods have paid attention to the characteristics of urban ecosystem health, the intrinsic uncertainty of urban ecosystem health is not fully emphasized. Intrinsic uncertainty is induced by the urban ecosystem’s features of openness, complexity and human-dominance. This negligence will impact the objectivity of the health assessment results.

Reviewing these methods of urban ecosystem health assessment based on different focuses, certain essential issues need to be considered. These issues include the development of (1) a holistic conceptual model that can organize multiple factors and represent the key features of urban ecosystems in terms of both external performance and internal characteristics, (2) a scientific method that can deal with the indicator data and improve the objectivity and practicability of assessment results, and (3) a systematic framework that can cover the basic elements of urban ecosystem health assessment and be used as a paradigm for describing urban ecosystem health in a certain stage.

Based on these concerns, a framework for urban ecosystem health assessment is proposed in Section 2, covering theoretical foundations, assessment methods and practical applications. Concrete methods in terms of external indicators, internal indicators and health standard are also established in Section 3. The practice of urban ecosystem health assessment in China is briefly introduced in Section 4 to illustrate the application of the proposed framework and methods. The main findings of urban ecosystem health assessment in China are summarized. Finally, some prospects of urban ecosystem health assessment are discussed in Section 5.

## 2. Framework for Urban Ecosystem Health Assessment

**Figure 1:**
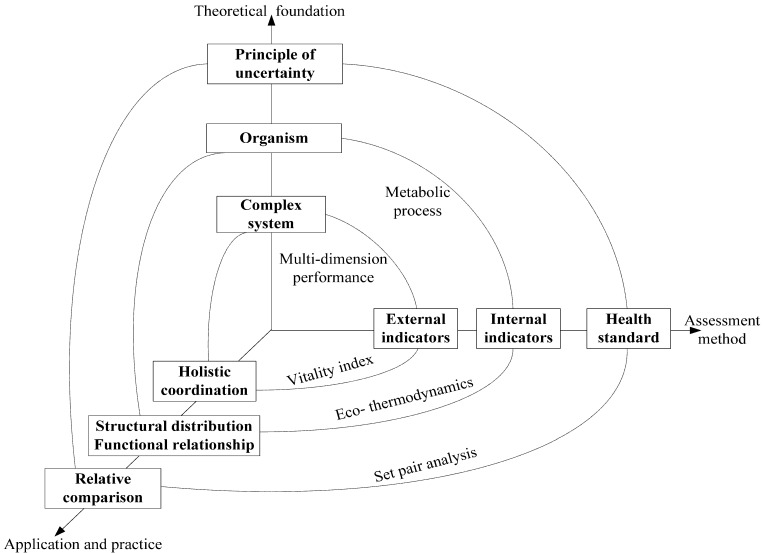
Fundamental framework of urban ecosystem health assessment.

Based on a review of the development of urban ecosystem health assessment [2], the essential elements were extracted and organized to form a fundamental framework for urban ecosystem health assessment, as shown in Figure 1. The framework for urban ecosystem health urban ecosystem health is represented by a triaxial cube that includes three dimensions: the theoretical foundation (see vertical axis), assessment method (see horizontal axis), and application and practice (see depth axis).

First (see the inner circle in Figure 1), urban ecosystems are complex systems composed of various subsystems (e.g., natural, economic, and social subsystems) and factors (e.g., air, water, and green areas in natural subsystem, industrial production and currency communication in economic subsystem, human living and culture exchange in social subsystem). To comprehensively assess the health state of urban ecosystems, multiple dimensions of performance must be considered simultaneously. On this basis, a vitality index integrating the external characteristics of different subsystems and the harmonious level functioning in these subsystems is established. This is done to incorporate the holistic coordination and optimization of the urban ecosystem into polices. This approach implies that such aspects as social civilization and environmental protection are as important as economic development.

Second (see the middle circle in Figure 1), the urban ecosystem can be regarded being similar to an organism, in which different components of the system are organized in a certain order and closely linked to each other by metabolic processes. In a healthy urban ecosystem, various components interact well, each metabolic procedure moves smoothly, and the whole system functions well. To check the internal metabolic situation of the urban ecosystem, eco-thermodynamic methods (e.g., emergy synthesis that links multiple factors in ecosystems connected by energy flows and unifies them by a common unit, where emergy is an expression of all the energy used in the work processes that generate a product or service in units of one type of energy) are developed based on which structural regulation and functional optimization can be addressed in urban management.

Third (see the outer circle in Figure 1), set pair analysis-a useful uncertainty method emphasizing the connections among different sets-is introduced to generate the health standard [32], when considering that the uncertainty of urban ecosystem health is derived from its intrinsic human value preferences-oriented characteristics [2]. This enables a relative comparison among different cities. The relative comparison is believed to be helpful for learning from other cities and improving the overall health levels of multiple cities.

Finally, in the three-dimensional cube, each pair of dimensions has a common interface, which means each dimension interacts with the other two dimensions. Based on the theoretical framework, the assessment methods are outlined. Then the assessment methods are applied to evaluate urban ecosystems and practical suggestions for urban regulation are offered based on the results of the analysis. Application of the theory and methods to compare urban ecosystems can be used to perfect the urban ecosystem health theory and assessment methods.

## 3. Methods of Urban Ecosystem Health Assessment

Based on the above framework, concrete methods of urban ecosystem health assessment are established. As shown in Table 1, three types of methods are included while each method has its own focus, characteristics, and progress.

**Table 1:** Methods of urban ecosystem health assessment.

Focus	Type	Method	Characteristics and progress
Indicators	External indicators	Vitality index model	Begins to integrate the characteristics of each subsystem and the harmony among urban subsystems when focusing on external performance of urban ecosystems.
	Internal indicators	Eco-thermodynamics synthesis	Starts to analyze the internal metabolic process based on unified accounting of various factors.
Standards	Relative standards	Set pair analysis	Generates relative health standards among multiple assessed objects.

**Figure 2:**
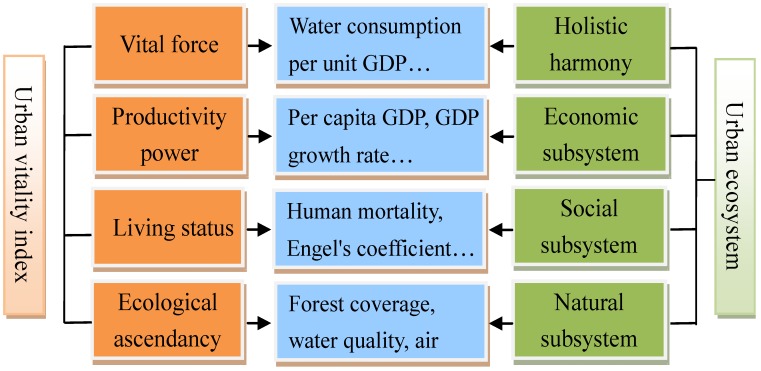
Sketch of the vitality index model of urban ecosystem health assessment.

**Figure 3:**
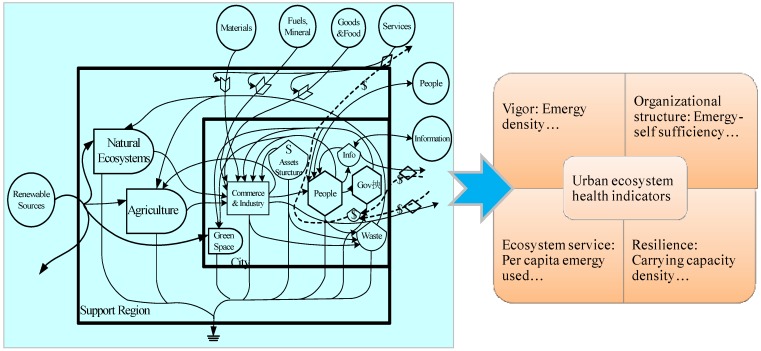
Sketch of the eco-thermodynamics model of urban ecosystem health assessment.

To understand the methods of urban ecosystem health assessment, some key points are explained as follows:

(1) The different methods have a common theoretical foundation. Urban ecosystems are similar to vital organisms composed of various subsystems and factors. In urban ecosystems, various factors have their own roles and must interact with each other by metabolic linkages to maintain the system’s structure and function. The health of each factor and smooth functioning among them are important for the holistic health status of urban ecosystems.

(2) Different methods are proposed in a specific order. The vitality index model (see Figure 2) was first put forward after understanding the natural-socioeconomic-interacted characteristics of the urban ecosystem. Natural, economic, and social subsystems were considered simultaneously, as well as the harmonious condition among these subsystems, to comprehensively evaluate the external performance of urban ecosystems. Eco-thermodynamic methods like emergy synthesis (see Figure 3) were then developed to analyze the internal metabolic processes, in which various types of energy and material flows were integrated and accounted in the same unit. Finally, returning to the essential problem of urban ecosystem health standards, set pair analysis was utilized to compare the health states of different urban ecosystems based on the generated standards. The relative comparison is believed to provide a distinct and understandable reference point for improving the health level of urban ecosystems.

(3) Each method plays a different role in the holistic system. Methods based on urban metabolism processes are regarded as the core of the whole system. They fully reflect the internal interaction among various factors and the holistic operation of urban ecosystems. The vitality index model focusing on external performance of urban ecosystems is an important supplement for the internal analysis. Set pair analysis aims at improving the objectivity of health standards and assessment results, which should be combined with other methods.

## 4. Practice of Urban Ecosystem Health Assessment in China

### 4.1. A Brief Introduction to Urban Ecosystem Health Assessment Using the Proposed Framework and Methods

The proposed framework and methods of urban ecosystem health assessment have been extensively applied to analyze many Chinese cities, e.g., Beijing, Chongqing, Guangzhou and other capitals [33,34,35,36], Fushun, Hangzhou, Xiamen and other medium-small cities [32]. In addition, urban ecosystem health assessments have been extended to other scales, including urban subsystems [24], urban clusters [37], and to a comparison among Chinese and foreign cities [38].

**Figure 4:**
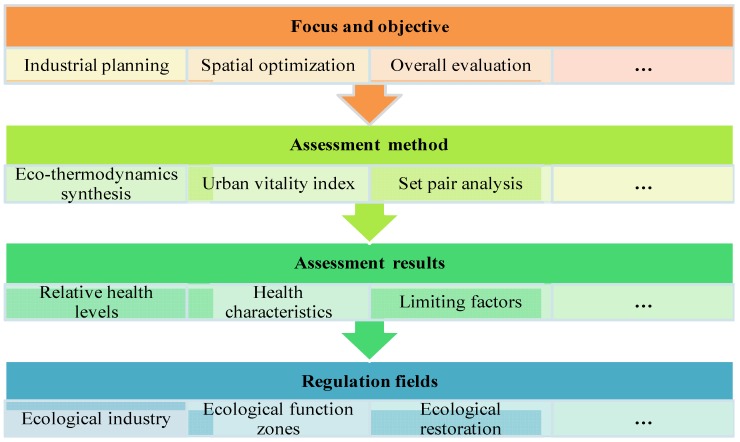
Procedure of urban ecosystem health assessment.

The basic procedure of urban ecosystem health assessment is summarized in Figure 4. To demonstrate the application of the proposed theoretical framework and methods of the urban ecosystem health assessment in more detail, three cases are briefly introduced in Table 2.

First, the urban ecosystem health assessment method is established following a concrete focus and objective. Eco-thermodynamics can be applied if the results of an urban ecosystem health assessment are needed for industrial adjustment and landscape planning. The vitality index can be utilized to roughly evaluate the external performance of urban ecosystems. Then, an urban ecosystem health assessment is conducted through which the concrete limiting factors of urban ecosystems are identified. Finally, the key regulation fields for the future are defined based on the limiting factors of the urban ecosystem.

**Table 2:** Urban ecosystem health assessment in three Chinese cities.

City	Characteristics	Focus and objective	Assessment methods	Regulation fields based on limiting factors
Beijing	The capital of China, having a burdensome population	Industrial planning based on internal metabolic processes	Eco-thermodynamics synthesis	Controlling population; developing ecological industry; and advocating resource saving
Guangzhou	A megacity located on the Pearl River serving as a commercial and financial center in South China	Spatial optimization based on internal metabolic processes	Eco-thermodynamics synthesis and set pair analysis	Controlling population; dividing ecological function zones and constructing landscape security pattern
Wanzhou, Chongqing	A migration city located on the upper reaches of the Three Gorges	Overall evaluation based on external performance	Urban vitality index and set pair analysis	Developing ecological economy; conducting ecological restoration and constructing human settlements

### 4.2. Main Findings of Ecosystem Health Assessment of Chinese Cities

Based on the extensive practice of urban ecosystem health assessment in China, some interesting findings are revealed. The findings are extensive, and three examples are provided below to illustrate the significance of these findings:

(1) Spatial distribution rule of the level of urban ecosystem health for Chinese cities.

By comparing the health levels of thirty-one Chinese cities, the method identified the arch-shaped distribution rule (by longitude) of the level of urban ecosystem health [34]. It revealed that the coastal cities (e.g., Shanghai, Hangzhou, Fuzhou, Guangzhou, *etc.*) and the frontier areas of inland China (e.g., Urumchi) are located on the same curve with a relatively low level of health. The coastal municipalities (e.g., Jinan, Wuhan, Changsha, Nanning, *etc.*) and some inland cities (e.g., Yinchuan, Lanzhou, *etc.*) are located on a curve with medium level of health. Other inland cities (e.g., Chongqing, Kunming, Chengdu, *etc.*) are located on another curve with relatively high levels of health. Over-development and over-concentration of urban areas in coastal regions cause great environmental pressure, which restrains healthy urban development. The poverty in northwest China results in relatively low level of health in urban ecosystems. It implies that economic development, resource exploitation and environmental protection should be addressed simultaneously to shape a healthy urban ecosystem.

(2) Classification of the health mode of urban ecosystems for Chinese cities.

Except for grading the health levels of different cities, the health modes of Chinese cities are classified based on their performance across multiple factors of urban ecosystem health. According to cluster analysis of eco-thermodynamics-based health performance, the thirty-one analyzed Chinese cities are classified into six groups according to urban ecosystem health status. The classifications include Shanghai mode, Lhasa mode, Changchun mode, Beijing mode, Xi’an mode and Chongqing mode [34]. The limiting factors of urban development differ with the health modes, e.g., cities in Shanghai mode are restricted by high environmental impact associated with high economic flux while cities in Xi’an mode are limited by low urbanization level [39]. Based on these modes and their main limiting factors, the idea of classified management needs to be highlighted in practice.

(3) Spatial differences of urban ecosystem health levels within an urban ecosystem.

In an urban ecosystem, the health levels of different subsystems also represent the character of spatial distribution. Taking Guangzhou as an example, the health levels of different land-use subsystems first rise from the north to the middle and south, and then decline from the middle and south to the southwest [24]. Based on the spatial differences in health status, the zoning management strategy should optimize structure and function in a holistic way. Therefore, the north of Guangzhou, an area mainly comprised of forests, grasslands, rivers, and wetlands, is defined as a conservation area. The middle and southern parts composed of cultivated land and general commercial areas are defined as maintenance areas where measures should be implemented to increase energy usage efficiency and maintain indigenous renewable resources. Finally, the south central and southwestern parts, mainly involving production and consumption centers and traffic areas, are defined as key regulation areas where more space should be converted for environmental-friendly living activities and efficient production, population should be controlled, energy demands should be reduced, and waste discharge should be decreased.

## 5. Discussion and Conclusions

A holistic framework for urban ecosystem health assessments is necessary to shed light on critical problems of urban ecosystem health. Problems include how to: (1) build a holistic conceptual model to systematically organize multiple factors, (2) establish a reasonable health standard, (3) improve the objectivity of assessment results, and (4) make the assessment results more constructive for practical urban management. Based on a review of urban ecosystem health assessment, a framework for urban ecosystem health assessments is proposed, in which the essential elements of urban ecosystem health are included and integrated using a model with three dimensions, theoretical foundation, assessment method and practical application.

For a person, the external health performance (e.g., complexion and vigor) and the internal biochemical character (e.g., enzymes and proteins) are both important for evaluation of health status. The former is easier and faster to observe and the latter is more accurate for confirming a diagnosis. Similarly, the external performance and internal characteristics are both important for an urban ecosystem in that they can represent the overall situation from different viewpoints. Therefore, assessment methods focused on external performance and internal metabolic processes are both crucial to an urban ecosystem health assessment. If different methods are applied at the same time, the assessment results should be checked carefully. Especially when the results based on different methods seem to be inconsistent with each other, the assessment procedure should be rechecked, because further analysis may be necessary to clarify the gap.

Urban ecosystems are a typical complex open system. They link closely with and are greatly influenced by their surroundings through energy and material flow, information circulation, and cultural communication. Each urban ecosystem has multiple roles at different layers with different functions [40]. For a given urban ecosystem, factors such as the relationship with adjacent cities and relative level of development will contribute to an understanding of its urban ecosystem health status, in addition to the internal situation of the urban ecosystem itself [41]. Therefore, urban ecosystem health assessments at multiple scales are necessary in the future. Through these assessments, the following can be achieved: more comprehensive understanding of urban ecosystem health, more accurate orientation of urban development, and more feasible programs for practical urban management.

A healthy urban ecosystem not only performs well in terms of structural stability and functional completeness under normal conditions, but it also has a strong ability to adapt and recover when facing change and even threat. Urban ecosystem health is relevant to future development potential. It is as important as the current health status, guided by the idea of sustainable development. Based on these dynamic characteristics of urban ecosystem health, assessments should be conceptualized as a process, combining with value-driven characteristics. Assessments will also give us more hope and impel us to focus more studies on the dynamic trends of health status. Consequently, assessment of urban ecosystem health status should be applied throughout urban ecological planning and management, including in status quo assessment of urban ecosystems, optimization of planning programs, and post-evaluation of a program’s effect.
